# Vas Deferens Abscess Rupture: A Case Report

**DOI:** 10.5334/jbsr.2840

**Published:** 2022-07-27

**Authors:** Beom Kyun Pak, Dong Min Kang, Sang Heon Kim

**Affiliations:** 1 Presbyterian Medical Center, KR

**Keywords:** multidetector computed tomography, lower urinary tract infections, vas deferens, abscess

## Abstract

A vas deferens abscess is a very rare complication of acute vasitis and lower urinary tract infection. A case of vas deferens rupture due to an abscess with severe pelvic inflammation requiring surgical drainage is reported.

**Teaching Point:** Vas deferens abscess rupture is an example of a very rare complication of severe inflammation of the vas deferens.

## Introduction

Abscess in the vas deferens (VD) is a very rare condition. Most VD inflammation is considered a retrograde infection from the lower urinary tract, that is, the urethra, prostate, seminal vesicles, and urinary bladder [1,2]. There is one case of VD abscess and several cases of spermatic cord abscess in the literature [3]. Here, we report a case of VD rupture due to an abscess, which is even rarer than these VD abscess cases.

## Case Report

**Figure 1:**
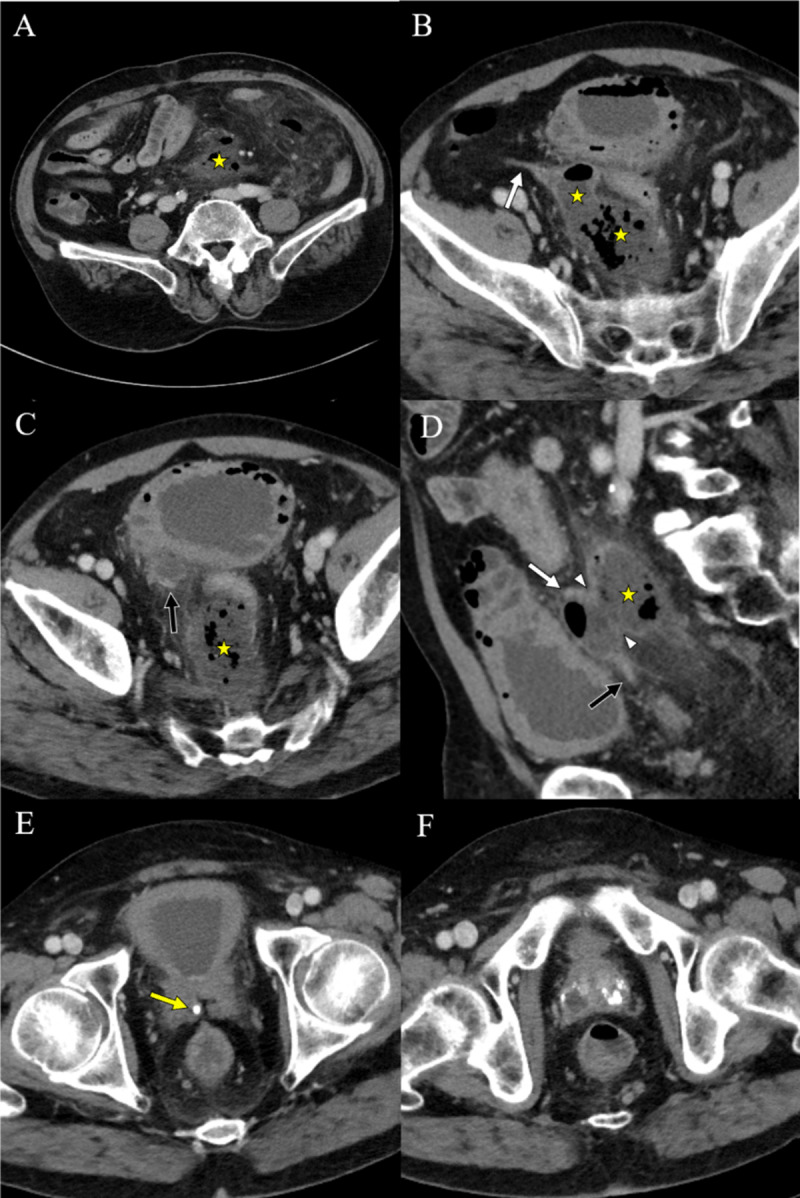
Pre-treatment CT images of a 65-year-old man with rupture of a vas deferens (VD) abscess. Axial and sagittal images **(A–D)** show a large abscess along the sigmoid mesocolon. Axial images **(B, C)** show the proximal and distal portion of right VD. A sagittal image **(D)** shows a wall defect of the VD adjacent to the abscess. An axial image **(E)** shows a tiny stone at the point where the VD joins the seminal vesicle. Axial images **(F)** show multifocal abscesses and dystrophic calcifications in the prostate. (asterisk: abscess, white arrow: proximal VD, black arrow: distal VD, arrowhead: VD wall defect, yellow arrow: VD stone).

**Figure 2:**
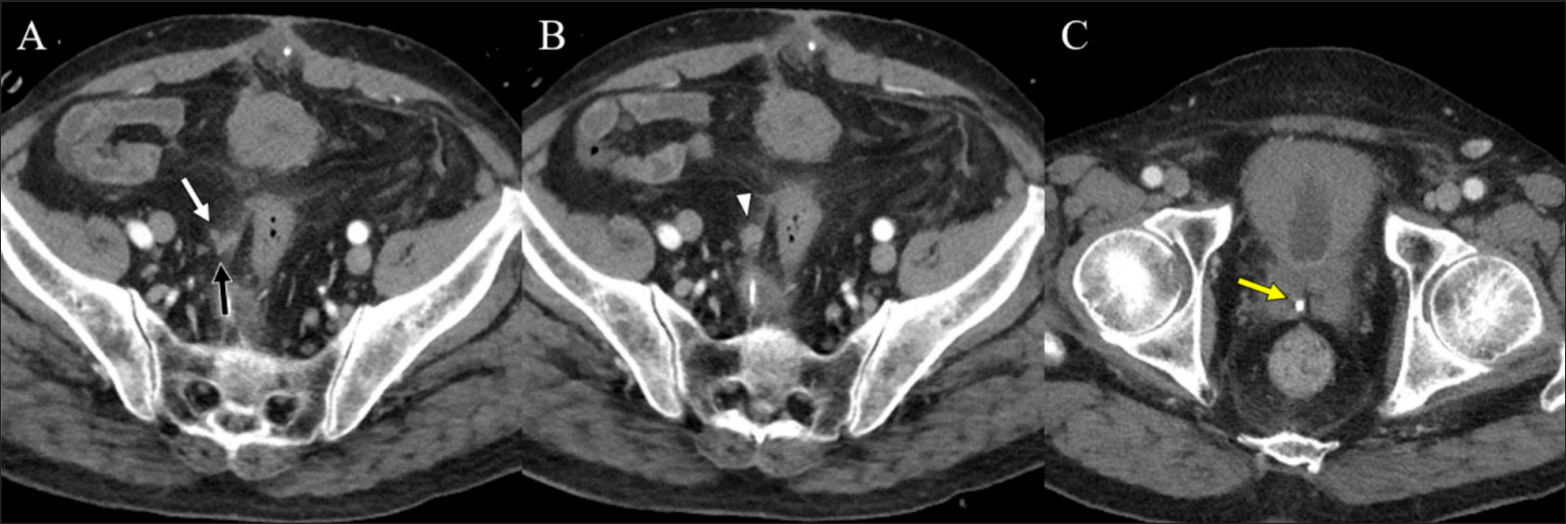
Follow-up CT images were taken after 11days. Axial images **(A, B)** show no abscess other than enhancing soft tissue (arrowhead) around the defect site of the VD wall. An axial image **(C)** shows a remnant stone at the distal VD. (white arrow: proximal VD, black arrow: distal VD, yellow arrow: VD stone).

A 65-year-old man was referred to the emergency department with fever and abdominal pain. He had a medical history of benign prostatic hyperplasia and neurogenic bladder. In laboratory tests, pyuria, leukocytosis, and an elevated serum C-reactive protein level were noted. A contrast-enhanced computed tomography (CT) scan showed a large abscess in the extraperitoneal space and the sigmoid mesocolon (Figure 1A–1D). A wall defect of the right VD adjacent to the abscess was observed in the retrospect (Figure 1D). A tiny stone was likely to be present in the right distal VD (Figure 1E). In addition, there was an abscess and multiple calcifications in the prostate (Figure 1F). A CT scan performed five years ago showed dystrophic calcifications in the prostate, but a distal VD stone was not present. Based on this imaging study, the patient underwent an abscess drainage operation. Unfortunately, during laparoscopic surgery the cause of the abscess was not recognized. Because the abscess was confined to extraperitoneal space and sigmoid mesocolon, the defect of the VD wall was not seen during surgery. Follow-up CT was performed 11 days later, and the abscess was completely resolved. There was some residual enhancing soft tissue around the estimated wall defect site of the VD (Figure 2A, 2B). The VD stone was still visible (Figure 2C). After three weeks of antibiotic treatment, the patient was discharged.

## Discussion

The VD is located in the extraperitoneal space and courses from the epididymis to seminal vesicle above the prostate. The VD may be affected by inflammation of adjacent organs [1]. The most common clinical diagnosis of inflammation in the VD is acute vasitis. The pathogenesis of acute vasitis is usually the result of the retrograde spreading of pathogens [2]. However, inflammation does not spread easily into the VD because the ejaculatory duct enters the verumontanum tangentially and serves as a valve to prevent the reflux flow into the VD [4].

In our case, there were dystrophic prostate calcifications. On previous imaging studies, there was no calcification near the distal end of the right VD. It is most likely that prostatitis was complicated with multifocal prostate abscesses. It is speculated that the tiny stone in the distal VD appeared to be a prostatic calcification migrated upward due to inflammatory weakening of the ejaculatory duct. It is important that this assumption is substantiated by the fact that at the time a bladder catheter was inserted at the emergency room, the catheter tip was located outside the prostatic urethra, indicating that there was already damage to the prostatic urethra. Inflammation from the prostate spread to the VD, with migration of prostatic calcification into the VD, which appears to have been predisposing for the VD rupture.

During initial reading of the CT study at the time of the admission to the emergency department, the VD rupture was not reported. A retroperitoneal abscess was reported, without clear origin, such as UTI or diverticulitis. Thus, in the process of carefully relooking for other causes, the VD rupture was observed.

To our knowledge, there is no previously reported case of VD rupture due to an abscess in the literature. We share a case of a very rare clinical experience of complicated VD rupture due to an abscess.
